# Supplementary material for: Pulses of ocean acidification at the Triassic–Jurassic boundary
Source: Nat Commun. 2025 Jul 14;16:6471. doi: 10.1038/s41467-025-61344-6 (PMC12260068; doi:10.1038/s41467-025-61344-6)
Supplement: Supplementary file 1 — Supplementary Information [file 41467_2025_61344_MOESM1_ESM.pdf]

Supplementary Information for

Pulse of ocean acidification at the Triassic-Jurassic boundary recorded by boron isotopes

Molly Trudgill, James W.B. Rae, Ross Whiteford, Markus Adloff, Jessica Crumpton-Banks, Michael Van Mourik, Andrea Burke, Marieke Cuperus, Frank Corsetti, Daniel Doherty, William Gray, Rosanna Greenop, Wei-Li Hong, Aivo Lepland, Andrew McIntyre, Noor Neiroukh, Catherine V. Rose, Micha Ruhl, David Saunders, Magali M.F.R. Siri, Robert C.J. Steele, Eva Stüeken, A. Joshua West, Martin Ziegler, Sarah E. Greene
Correspondence to: molly.trudgill@lsce.ipsl.fr

Supplementary Text

Bulk rock $\delta^{11}B$

Finding a stratigraphic section where a single species spans an extinction horizon is difficult, hindering the use of the $\delta^{11}B$ proxy across such intervals. Indeed, no oysters are preserved across the extinction horizon at Lavernock Point and no other well-preserved carbonate-producing species are present across the entire extinction interval at this locality. Therefore, we explored using bulk rock $\delta^{11}B$ analyses to extend the record across the extinction itself. Bulk rock $\delta^{11}B$ has been used previously to reconstruct ocean pH in deep time (e.g., [1,2]), but the extent to which this proxy represents a primary environmental signature is debated (e.g. [3]). In particular, the presence of diagenetic features in Triassic–Jurassic age sections around the UK may indicate carbonate cementation driven by organoclastic sulphate reduction under very different porewater pH conditions than the overlying seawater [4–6]. Furthermore, the bulk rock may be comprised of several phases that, when the bulk rock is digested as a whole, this complicates the extraction of the primary geochemical signal. These different phases may have different solubilities, so chemically separating them with a sequential leach approach allows them to be measured independently. At Lavernock Point we collected paired bulk samples with the oyster samples. We used a sequential leaching method[7] to separate phases with different solubilities. Briefly, this involved an oxidative clean with 3 % hydrogen peroxide, 0.1M sodium hydroxide and a buffer clean with 0.1M ammonium acetate, followed by leaching in sequentially higher concentrations of acetic acid, from 0.25 % HAc up to 10 % HAc. The trace element of these bulk leach samples was analysed by quadrupole ICP-MS (Fig. S12). The boron (B) in these leaches was separated from the matrix as for the oysters, using the column chemistry procedure of Foster[8], and analysed for boron isotope composition the Neptune MC-ICPMS at the University of St Andrews following the procedures of Rae *et al.*[9]. By sequentially leaching the bulk rock, phases with different solubilities will dissolve at different points and can be measured independently, with the primary carbonate phase expected to dissolve in the earlier leaches, while secondary/clay phases predicted to be more resistant to dissolution and present in the later dissolution stages.

To obtain the boron isotope data from phases most likely carrying the primary signature, we measured the first, second, third and fifth leaches for $\delta^{11}B$ representing roughly 5, 10, 15 and 30 % $CaCO_3$ leached respectively (Fig. S13). All these bulk leaches show the same trend, with $\delta^{11}B$ increasing through the section from 1.5-3 ‰ at the base of the section, up to 6.5-10 ‰ towards the top of the stratigraphy. Both the absolute values and the trend in these bulk samples are completely different from the oyster $\delta^{11}B$ record (Fig. S13). This result suggests that none of the leaches from the bulk record preserve the primary $\delta^{11}B$ signature. This is likely the result of

micrite formation in shallow diagenetic settings. While the oysters make their shells from low-Mg calcite, which is relatively resistant to diagenesis (e.g., [10,11]), it is possible the bulk micrite is overprinted by diagenesis; there are certainly diagenetic features present in the section (nodular concretions, 'beef' rock e.g., [4,12]). Alternatively, the bulk carbonate may have never recorded seawater pH if it formed in porewaters where processes like pyrite formation and sulphate reduction altered porewater pH [4–6]. What the bulk $\delta^{11}B$ record does tell us is that the oysters are preserving a signal distinct from the bulk rock, and while it cannot conclusively prove that the oysters are preserving a primary signature, it supports that conclusion and shows that these units have not been completely homogenized by diagenesis and lithification.

Supplementary figures and tables

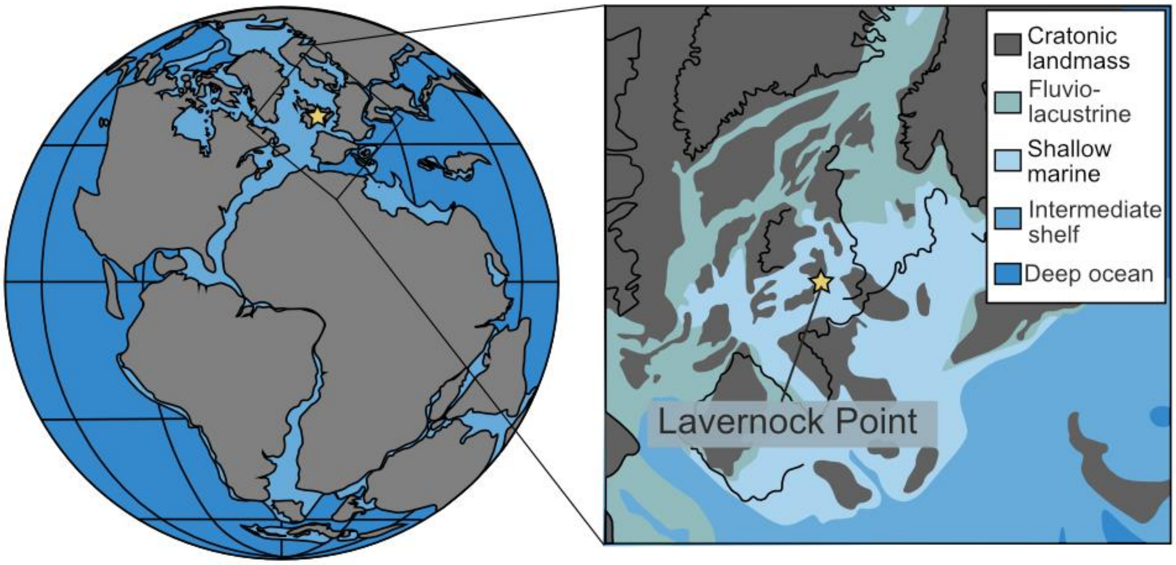

Fig. S1. Continent position, paleogeography and sampling location.

Outlines based on Ruiz-Martinez *et al.*[13] and Van de Schootbrugge *et al.*[14]

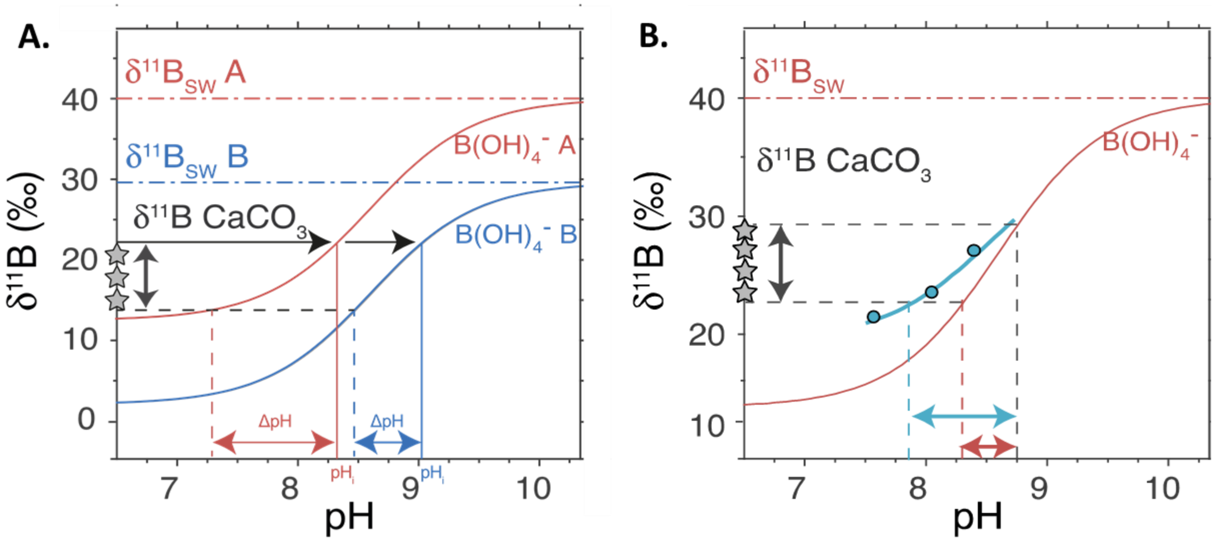

Fig. S2. Relationship between δ^{11}B and pH.

(**A**) The relationship between δ^{11}B and pH for borate ion at different δ^{11}B$_{SW}$. δ^{11}B$_{SW}$ controls the absolute pH calculated from measured δ^{11}B (grey stars), which will also control the ΔpH calculated due to the non-linear relationship between δ^{11}B and pH. At a lower absolute pH the gradient between δ^{11}B and pH is shallower, so the same change in δ^{11}B will represent a larger change in pH; the smallest ΔpH is in the centre of the curve (pH 8.6) where the gradient is steepest. (**B**) The impact of a hypothetical species calibration with a shallower gradient (light blue) than that of borate ion on absolute pH (dashed vertical lines) and ΔpH (arrows) calculated from a δ^{11}B record (grey stars). All currently measured species calibrations have gradients ≤1. A lower gradient will lead to a larger change in pH for the same δ^{11}B excursion.

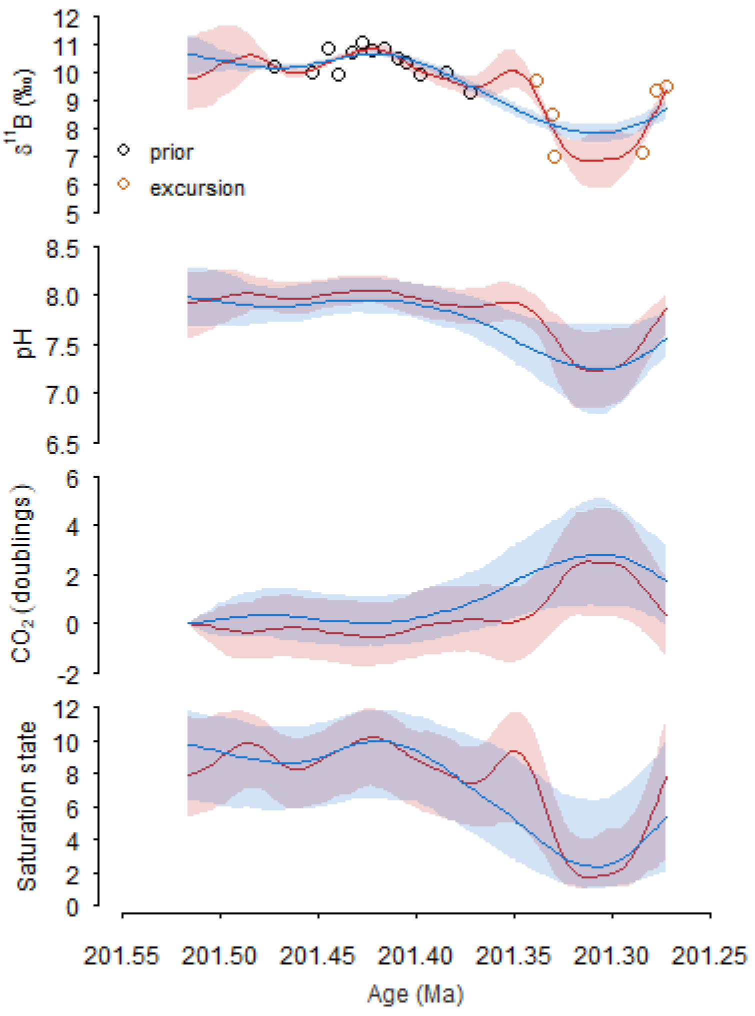

Fig. S3. Data with standard 18 kyr smoothing window used in main text/Figure 2 (red) and a smoother 50 kyr fit (blue). Shaded region represents 95% confidence interval. With a smoother fit although the shape of the record changes, the magnitude of change remains similar. ANOVA analysis of data prior to (black points) and covering the excursion (brown points) shows a statistically significant difference ($p = 0.0002$).

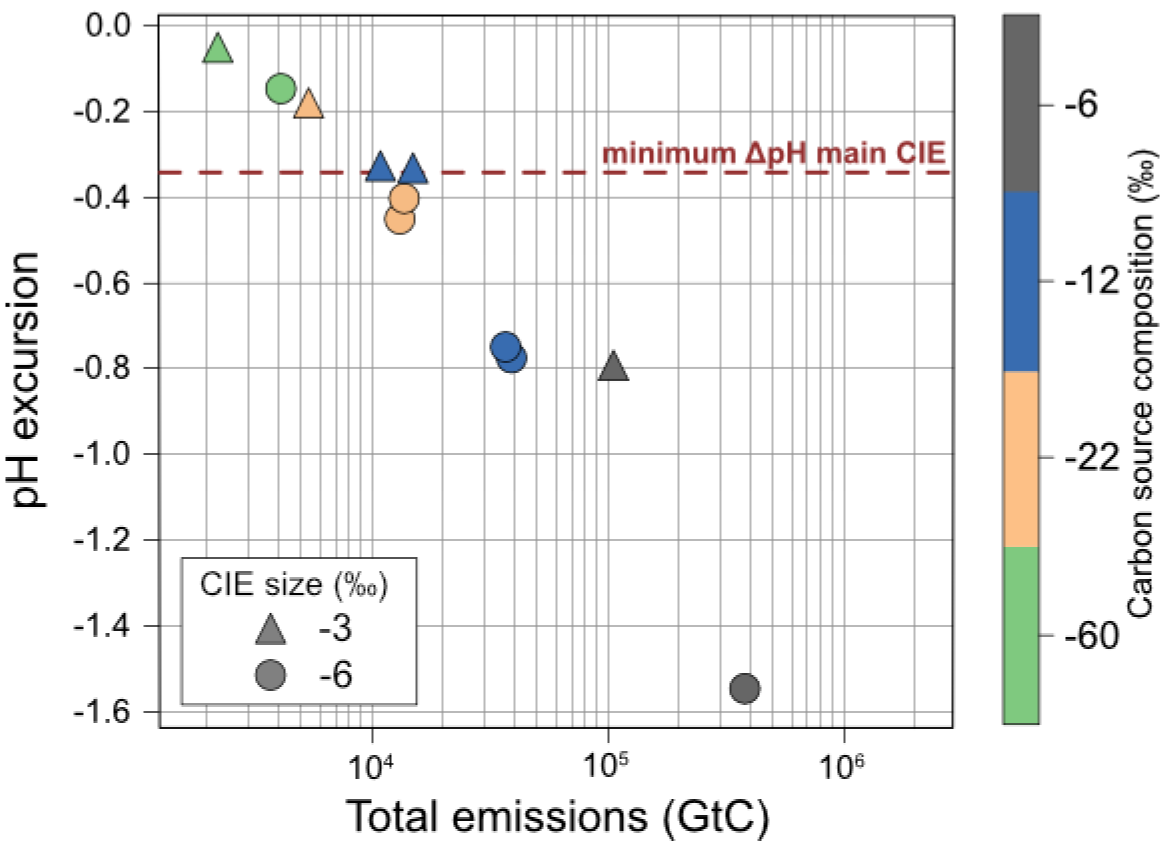

Fig. S4. Total emissions modelled to drive a 3 or 6 per mil carbon isotope excursion by a range of carbon sources. Modelling in cGENIE. Scenarios with a 75 kyr onset are plotted. Note that only the carbon isotope excursion (CIE) amplitudes explicitly modelled in the Vervoort et al.[15] simulation set are shown (-3 and -6 ‰). Interpolating these results to the CIEs of the Triassic-Jurassic (-2.2 and -5 ‰) and our reconstructed pH change gives emissions around 10-20,000 GtC, which is in line with independent CAMP emission reconstructions. Carbon releases from the CAMP alone are 30,000 – 50,000 Gt C[16,17] with sedimentary contributions estimated to be 75,000-88,000 Gt C[16,18]. Dashed red line indicates the minimum estimate for pH change across the main CIE.

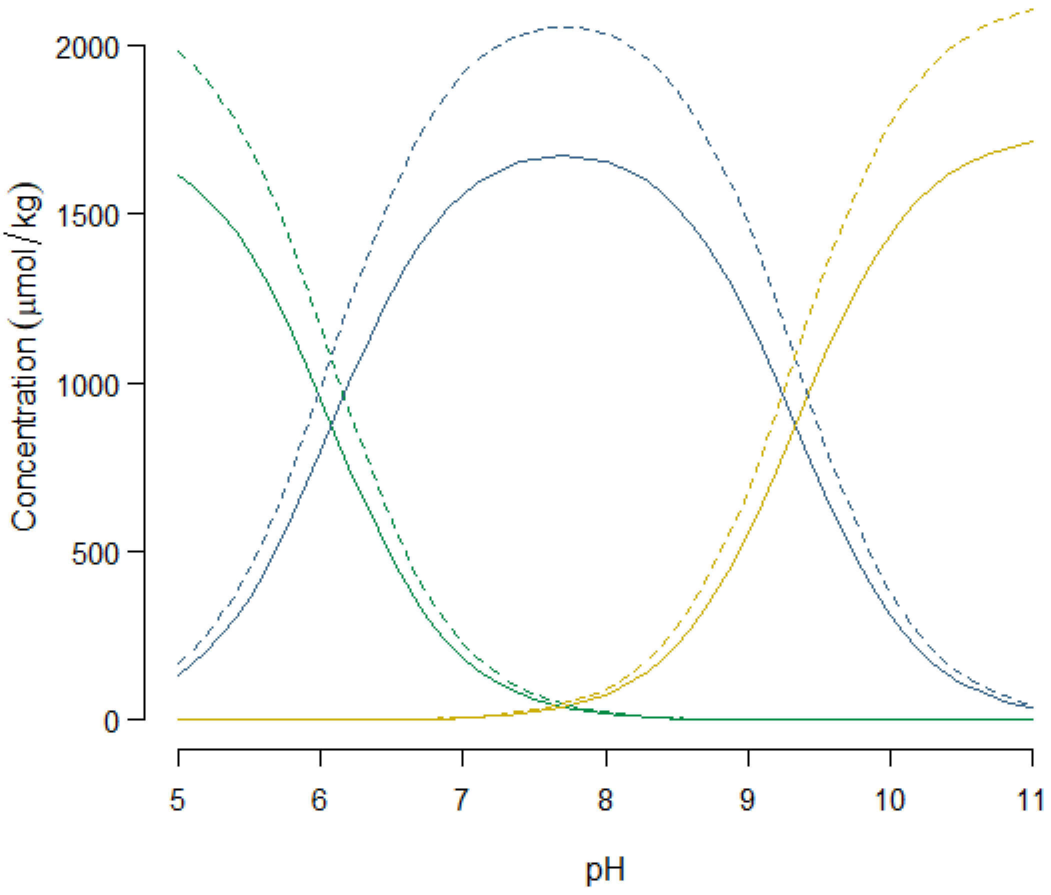

Fig. S5. Bjerrum plot showing carbonate speciation at a range of pH with a higher dissolved inorganic carbon (DIC) representative of the pre-pelagic ocean (2150 µmol/kg) and a lower DIC representing the ocean after the diversification of pelagic calcifiers (1750 µmol/kg). With a higher DIC the same proportion of carbonate ion is respeciated for a given change in pH, but the absolute magnitude of carbonate ion change – and thus change in saturation state – is greater.

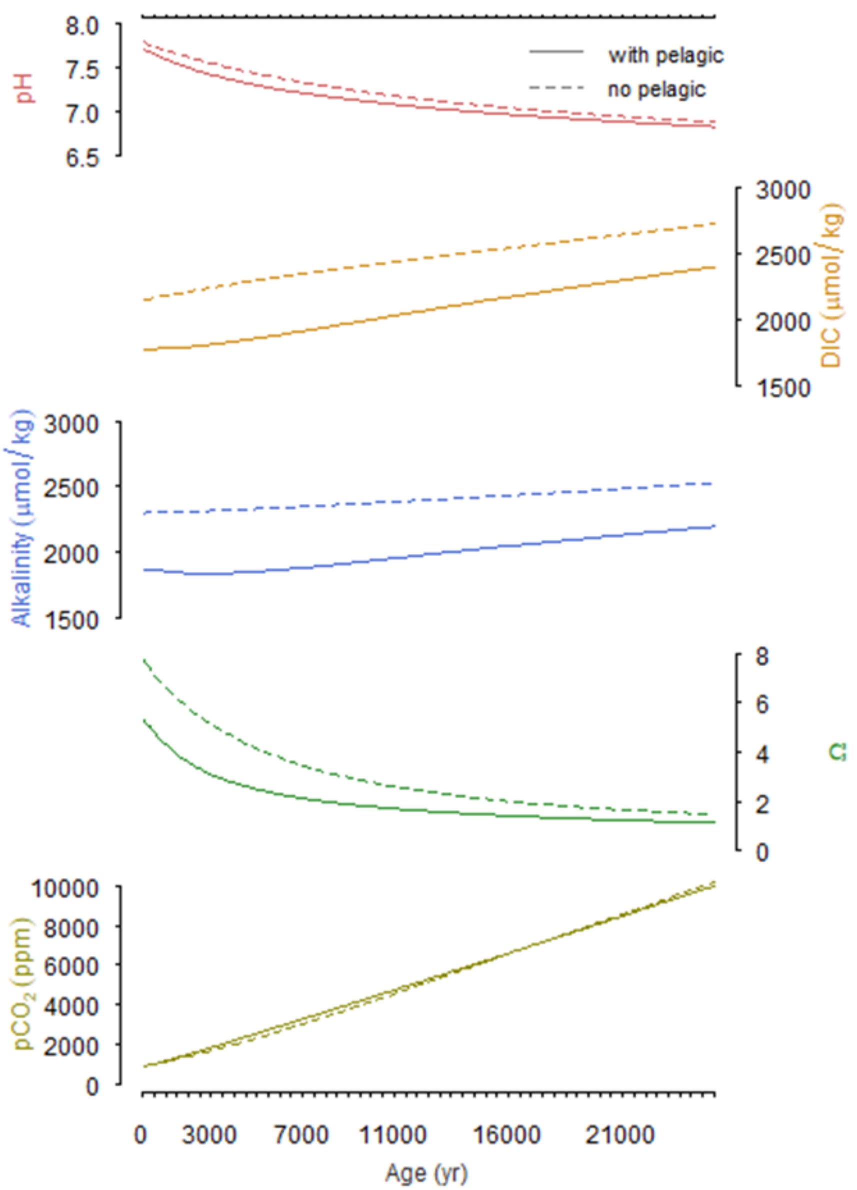

Fig. S6. Modelled carbonate system response to 1Pg/yr carbon injection in cGENIE both with (solid lines) and without (dashed lines) the presence of pelagic calcifiers. Neither model run includes climate driven weathering changes. While both show a similar pH decline for the same carbon input, the experiment without pelagic calcifiers shows a greater decrease in saturation state.

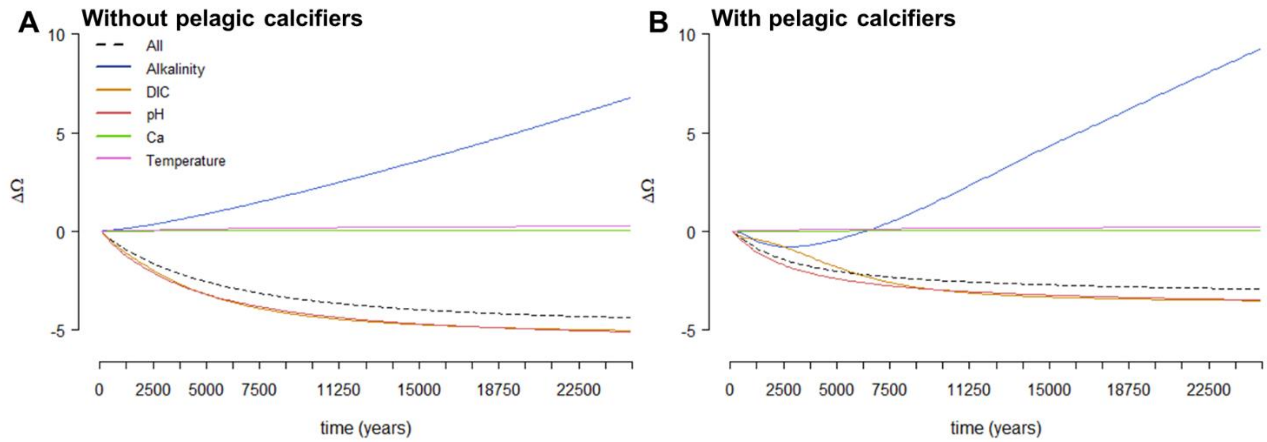

Fig S7. Drivers of change in saturation state for two model runs perturbed with 1PgC/yr, simulating conditions before (A) and after (B) the expansion of pelagic calcifiers. Weathering is held constant in these experiments to isolate oceanic processes. Coloured lines show the change in saturation state in an offline carbonate system calculation that individually changes alkalinity, dissolved inorganic carbon (DIC), pH, [Ca^{2+}] and temperature according to their cGENIE simulated excursions, while holding the others constant at pre-excursion values. Changes in [Ca^{2+}] and temperature have minimal influence on saturation state excusion of this scale, which is dominantly driven by addition of DIC and associated pH change. Saturation decline is somewhat buffered by increasing alkalinity, the result of reduced $CaCO_3$ burial relative to weathering input. In the scenario with pelagic calcifiers there is an initial decrease in alkalinity due to a reduction in export production and thus a reduction in water column $CaCO_3$ dissolution, likely driven by nutrient limitation due to warming-induced stratification.

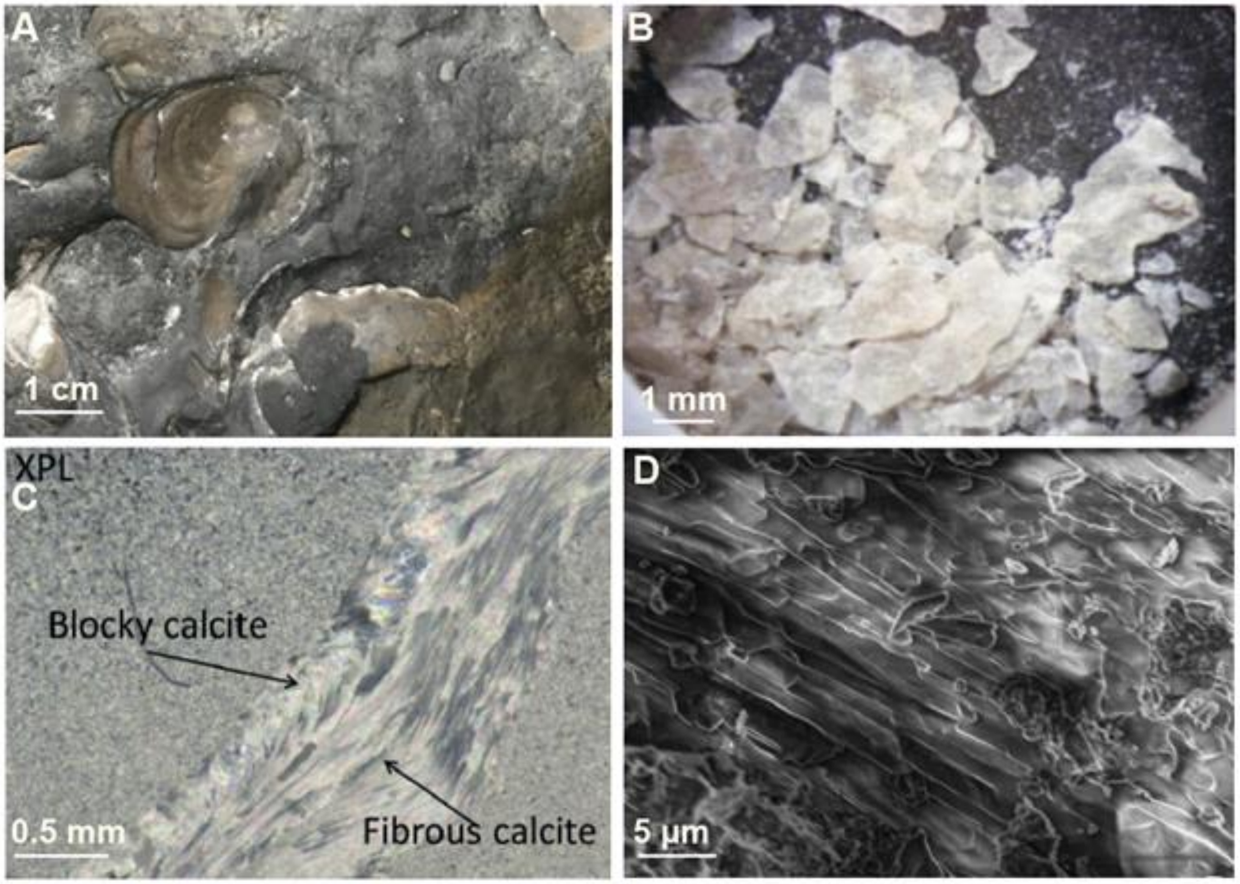

Fig. S8. Sample preservation

(**A**) Fossil oyster *Liostrea hisingeri* in field. (**B**) Flaked oyster sample prior to cleaning. (**C**) A thin section through a fossil oyster in cross-polarised light. (**D**) Scanning Electron Microscope (SEM) image of oyster flakes. Images show original shell textures and foliations suggesting excellent preservation.

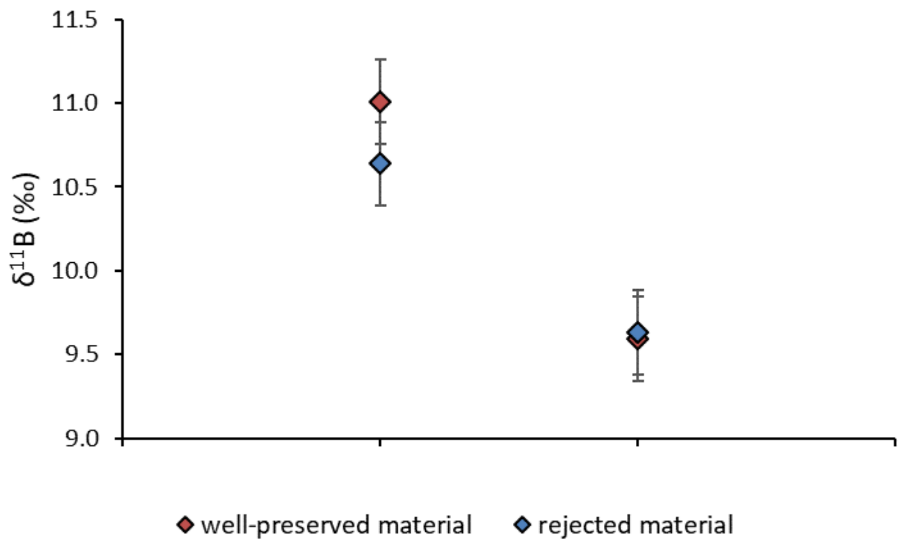

Fig. S9. Comparison of $\delta^{11}B$ measured for optically pristine and rejected material within the same sample. While optically pristine material and rejected material was largely within error, it's possible this may not always be the case, so only pristine material was taken for analysis. Error bars are 2SD.

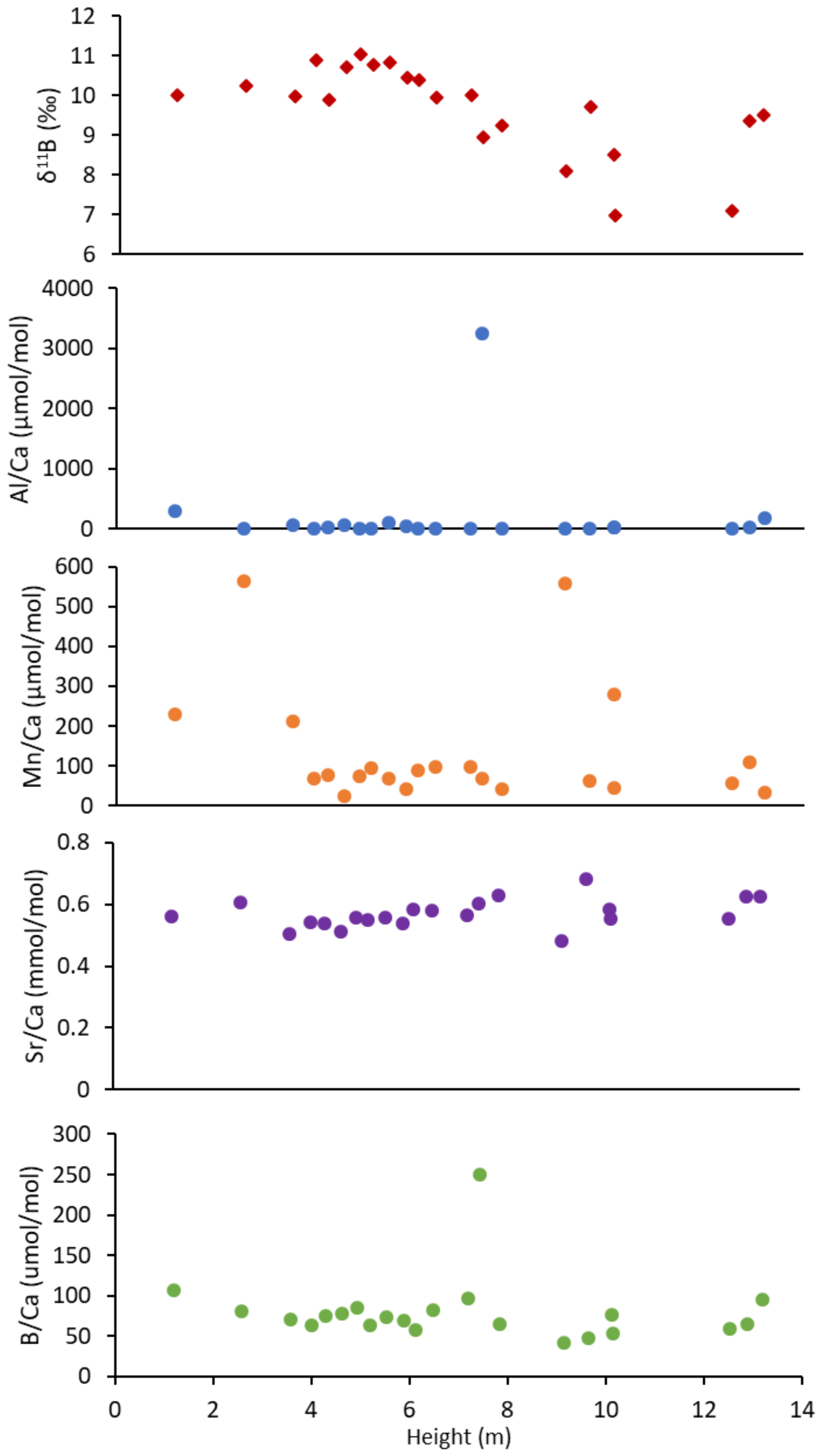

12
11
10
9
8
7
6
δ11B (‰)
4000
3000
2000
1000
0
Al/Ca (µmol/mol)
600
500
400
300
200
100
0
Mn/Ca (µmol/mol)
0.8
0.6
0.4
0.2
0
Sr/Ca (mmol/mol)
300
250
200
150
100
50
0
B/Ca (umol/mol)
0
2
4
6
8
10
12
14
Height (m)

Fig. S10. Comparison of δ^{11}B measured with selected trace elements which may indicate anomalous diagenetic conditions (low Sr/Ca, high Mn/Ca) or clay contamination (high Al/Ca).

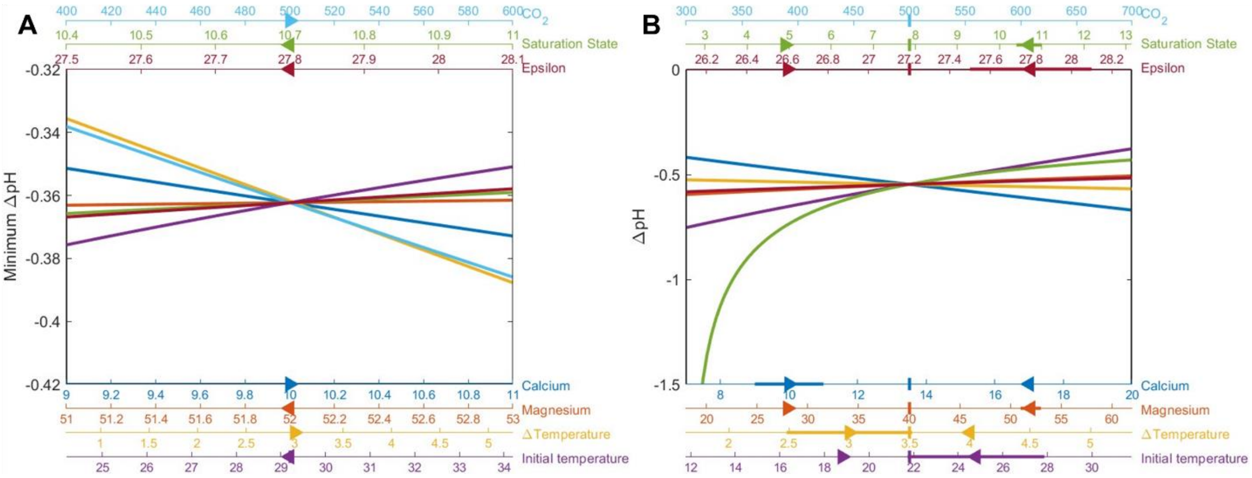

Fig. S11. pH sensitivity to input parameters.

Sensitivity of minimum change in pH (**A**) and change in pH (**B**) to initial CO_2 (ppm), initial saturation state, initial temperature (°C), Ca concentration (mmol/kg), Mg concentration (mmol/kg) and change in temperature over the duration of the δ^{11}B excursion (°C)

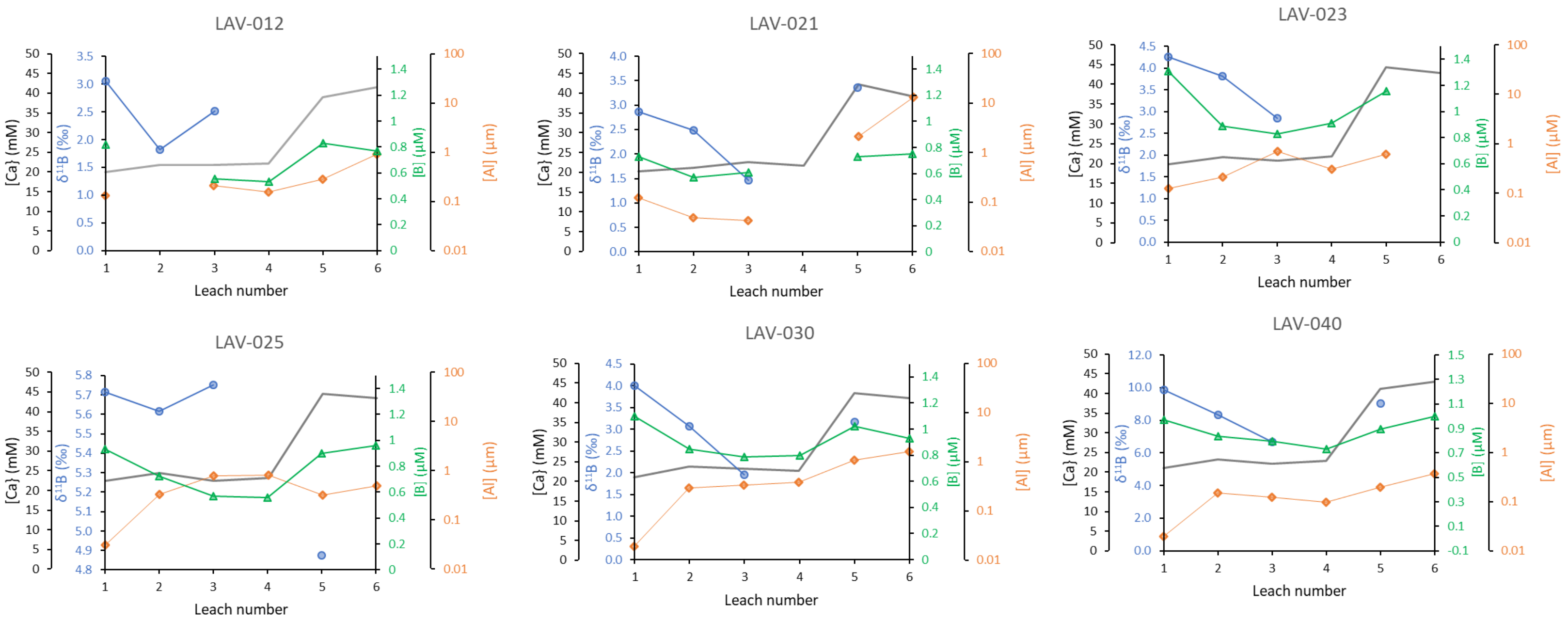

Fig. S12. δ^{11}B, Ca, B and Al concentration in each sequential leaching step for bulk leach samples. Increased leaching of clays in later sequential leaching steps may result in addition of isotopically depleted boron from clay surfaces, resulting in a general increase in Al and decrease in δ^{11}B with progressive leaching.

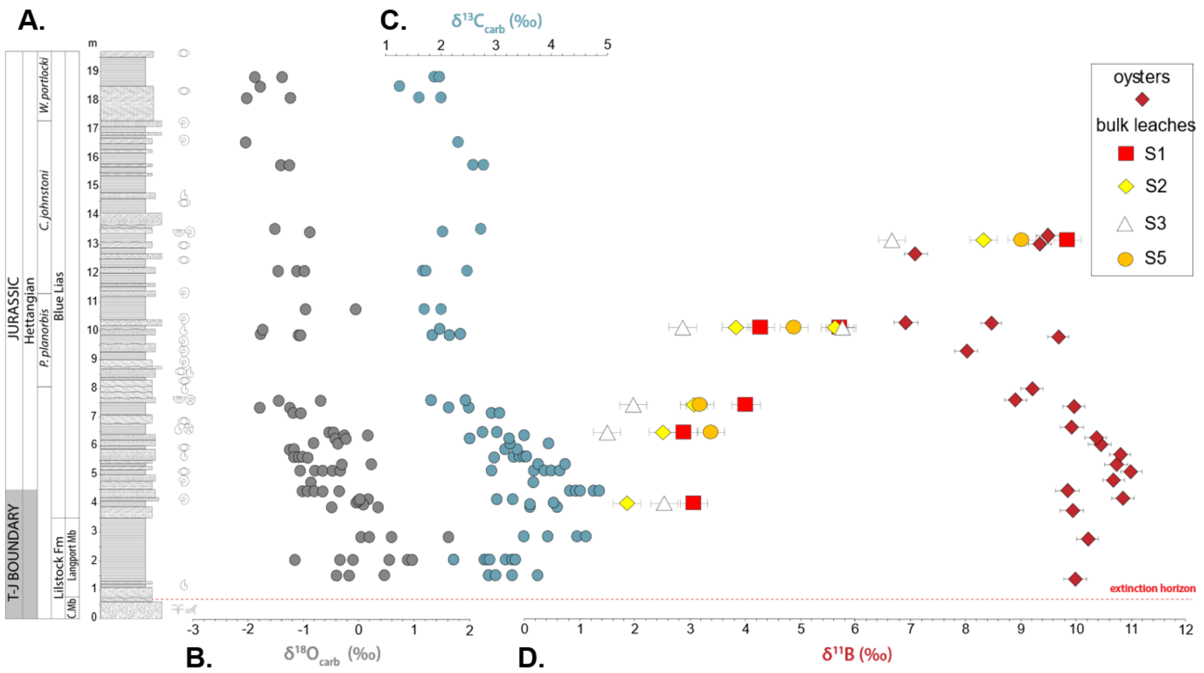

Fig. S13. Overview of lithological and geochemical data from Lavernock Point with bulk rock δ^{11}B.

Stratigraphy logged by the authors (**A**), δ^{18}O (**B**)[17], δ^{13}C (**C**)[17] and δ^{11}B (**D**) measured in the fossil oyster *Liostrea hisingeri* at Lavernock Point (red diamonds), compared to δ^{11}B measured in bulk rock leached sequentially in acetic acid following the methods of Hong *et al.*[7]. First (red squares), second (yellow diamonds), third (white triangles) and fifth (orange circles) acetic leaches shown. Error is long term external error based on repeated measurements of standards run with the same method in the STAiG lab (± 0.25 ‰).

Table S1. Parameters used for reconstruction of pH and atmospheric CO_2 from $\delta^{11}B$.

**Parameter**	**Minimum pH Change**	**Full uncertainties**	**Reference**
Initial CO_2	500 ± 50 ppm (minimum) (Uncertainty estimated)	500-5000 ppm (Upper end poorly constrained)	Witkowski *et al.*[19] McElwain *et al.*[20] Schaller *et al.*[21,22]
Initial saturation state	10.7 ± 0.15 (maximum) (Uncertainty estimated)	5.0-10.7	Ridgwell[23]
Calcium	8-10 mmol/kg (minimum)	8-17 mmol/kg	Horita *et al.*[24]
Magnesium	50-52 mmol/kg (maximum)	28-52 mmol/kg	Horita *et al.*[24]
Epsilon	28.4 ± 0.3 ‰ (maximum)	27.2 ± 0.6 ‰ or 26.0 ± 1.0 ‰	Klochko *et al.*[25] Nir *et al.*[26]
Species calibration gradient	1	0.25-1	Jurikova *et al.*[27] Penman *et al.*[28]
Species calibration intercept	0	-4-12	Jurikova *et al.*[27] Penman *et al.*[28] Sutton *et al.*[29]
Salinity	35 psu	35 psu	
Pressure (hydrostatic)	0 bar	0 bar	
Pressure (atmospheric)	1 atm	1 atm	

Table S2. Carbon cycle metrics in the cGENIE steady states with pelagic carbonate burial (the Vervoort et al. spin-up) and neritic carbonate burial.

	Pelagic carbonate burial	Neritic carbonate burial
Mean surface calcite saturation state	5.5	7.9
Mean surface $\delta^{13}C$ (‰)	3.07	3.07
Pelagic POC export (GtC/yr)	7	7
Pelagic $CaCO_3$ export (GtC/yr)	1.4	0.07
Pelagic $CaCO_3$ preservation (GtC/yr)	0.14	0.0
Neritic $CaCO_3$ preservation (GtC/yr)	0.0	0.09
$CaSiO_3$ weathering (TmCa/yr)	6	3
$CaCO_3$ weathering (GtC/yr)	0.072	0.052
CO_2 outgassing (GtC/yr)	0.072	0.035

Supplementary References

1. Kasemann, S. A., Prave, A. R., Fallick, A. E., Hawkesworth, C. J. & Hoffmann, K.-H. Neoproterozoic ice ages, boron isotopes, and ocean acidifi cation: Implications for a snowball Earth. *Geology* **38**, 775–778 (2010).
2. Clarkson, M. O. *et al.* Ocean acidification and the Permo-Triassic mass extinction. *Science (1979)* **348**, 229–232 (2015).
3. Clapham, M. E. & Renne, P. R. Flood Basalts and Mass Extinctions. *Annu Rev Earth Planet Sci* **47**, 275–303 (2019).
4. Bottrell, S. & Raiswell, R. Primary versus diagenetic origin of Blue Lias rhythms (Dorset, UK): evidence from sulphur geochemistry. *Terra Nova* **1**, 451–456 (1989).
5. Greene, S. E., Bottjer, D. J., Corsetti, F. A., Berelson, W. M. & Zonneveld, J. P. A subseafloor carbonate factory across the Triassic-Jurassic transition. *Geology* **40**, 1043–1046 (2012).
6. Soetaert, K., Hofmann, A. F., Middelburg, J. J., Meysman, F. J. R. & Greenwood, J. The effect of biogeochemical processes on pH. *Mar Chem* **105**, 30–51 (2007).
7. Hong, W. L., Lepland, A., Kirsimäe, K., Crémière, A. & Rae, J. W. B. Boron concentrations and isotopic compositions in methane-derived authigenic carbonates: Constraints and limitations in reconstructing formation conditions. *Earth Planet Sci Lett* **579**, 117337 (2022).
8. Foster, G. L. Seawater pH, pCO2 and [CO2−3] variations in the Caribbean Sea over the last 130 kyr: A boron isotope and B/Ca study of planktic foraminifera. *Earth Planet Sci Lett* **271**, 254–266 (2008).
9. Rae, J. W. B., Foster, G. L., Schmidt, D. N. & Elliott, T. Boron isotopes and B/Ca in benthic foraminifera: Proxies for the deep ocean carbonate system. *Earth Planet Sci Lett* **302**, 403–413 (2011).
10. Veizer, J. *et al.* Oxygen isotope evolution of Phanerozoic seawater. *Palaeogeogr Palaeoclimatol Palaeoecol* **132**, 159–172 (1997).
11. Veizer, J. *et al.* 87Sr/86Sr, d13C and d18O evolution of Phanerozoic seawater. *Chem Geol* **161**, 59–88 (1999).
12. Hallam, A. A sedimentary and faunal study of the Blue Lias of Dorset and Glamorgan. *Philosophical Transactions of the Royal Society B* **242**, (1960).
13. Carlos Ruiz-Martínez, V., Torsvik, T. H., Van Hinsbergen, J. J. & Gaina, C. Earth at 200 Ma: Global palaeogeography refined from CAMP palaeomagnetic data. *Earth Planet Sci Lett* **331–332**, (2012).
14. van de Schootbrugge, B. *et al.* Floral changes across the Triassic/Jurassic boundary linked to flood basalt volcanism. *Nat Geosci* **2**, 589–594 (2009).
15. Vervoort, P., Adloff, M., Greene, S. E. & Kirtland Turner, S. Negative carbon isotope excursions: an interpretive framework. *Environmental Research Letters* **14**, 085014 (2019).
16. Jiang, Q. *et al.* Volume and rate of volcanic CO2 emissions governed the severity of past environmental crises. *Proc Natl Acad Sci U S A* **119**, (2022).
17. Capriolo, M. *et al.* Deep CO2 in the end-Triassic Central Atlantic Magmatic Province. *Nat Commun* **11**, 1–11 (2020).
18. Heimdal, T. H. *et al.* Large-scale sill emplacement in Brazil as a trigger for the end-Triassic crisis. *Sci Rep* **8**, 141 (2018).

19. Witkowski, C. R., Weijers, J. W. H., Blais, B., Schouten, S. & Sinninghe Damsté, J. S. Molecular fossils from phytoplankton reveal secular PCO2 trend over the phanerozoic. *Sci Adv* **4**, eaat4556 (2018).
20. McElwain, J. C., Beerling, D. J. & Woodward, F. I. Fossil Plants and Global Warming at the Triassic-Jurassic Boundary. *Science (1979)* **285**, 1386–1390 (1999).
21. Schaller, M. F., Wright, J. D. & Kent, D. V. Atmospheric PCO2 perturbations associated with the central atlantic magmatic province. *Science (1979)* **331**, 1404–1409 (2011).
22. Schaller, M. F., Wright, J. D., Kent, D. V. & Olsen, P. E. Rapid emplacement of the Central Atlantic Magmatic Province as a net sink for CO 2. *Earth Planet Sci Lett* **323–324**, 27–39 (2012).
23. Ridgwell, A. A Mid Mesozoic Revolution in the regulation of ocean chemistry. *Mar Geol* **217**, 339–357 (2005).
24. Horita, J., Zimmermann, H. & Holland, H. D. Chemical evolution of seawater during the Phanerozoic: Implications from the record of marine evaporites. *Geochim Cosmochim Acta* **66**, 3733–3756 (2002).
25. Klochko, K., Kaufman, A. J., Yao, W., Byrne, R. H. & Tossell, J. A. Experimental measurement of boron isotope fractionation in seawater. *Earth Planet Sci Lett* **248**, 276–285 (2006).
26. Nir, O., Vengosh, A., Harkness, J. S., Dwyer, G. S. & Lahav, O. Direct measurement of the boron isotope fractionation factor: Reducing the uncertainty in reconstructing ocean paleo-pH. *Earth Planet Sci Lett* **414**, 1–5 (2015).
27. Jurikova, H. *et al.* Boron isotope systematics of cultured brachiopods: Response to acidification, vital effects and implications for palaeo-pH reconstruction. *Geochim Cosmochim Acta* **248**, 370–386 (2019).
28. Penman, D. E., Hönisch, B., Rasbury, E. T., Hemming, N. G. & Spero, H. J. Boron, carbon, and oxygen isotopic composition of brachiopod shells: Intra-shell variability, controls, and potential as a paleo-pH recorder. *Chem Geol* **340**, 32–39 (2013).
29. Sutton, J. N. *et al.* δ11B as monitor of calcification site pH in divergent marine calcifying organisms. *Biogeosciences* **15**, 1447–1467 (2018).
